# Characterization of airway mucus using quantitative MRI: an *in vitro* proof-of-concept study

**DOI:** 10.1186/s41747-026-00842-8

**Published:** 2026-10-26

**Authors:** Sam Sedaghat, Jin Il Park, Eddie Fu, Misagh Piran, Ahmadreza Ghasemiesfe, Youngkyoo Jung, Hyungseok Jang

**Affiliations:** 1 Department of Radiology, University Hospital Heidelberg, Heidelberg, Germany; 2 Department of Radiology, University of California, Davis, Sacramento, CA USA

**Keywords:** Lung, Magnetic resonance imaging, Mucin, Mucus, Pulmonary disease (chronic obstructive)

## Abstract

**Objectives:**

A characteristic of chronic obstructive pulmonary disease is mucus hypersecretion. In this proof-of-concept study, we aimed to characterize airway mucus using a quantitative ultrashort echo time (UTE) magnetic resonance imaging (MRI) sequence in a controlled *in vitro* synthetic mucus model to establish a technical foundation for future *in vivo* studies.

**Materials and methods:**

UTE double-echo steady-state (UTE-DESS) was performed to acquire the signal from free induction decay-like signal and spin/stimulated echo-like signal echoes; quantitative MRI parameters (T1, T2, and diffusivity) were estimated. Synthetic mucus phantoms were prepared using mucin (0.20%, 0.50%, and 1.0%) mixed with xanthan gum (0.10%, 0.25%, and 0.50%) to replicate the viscoelastic properties of human laryngeal mucus, and samples were embedded in 1% agar for imaging.

**Results:**

The mucus phantoms with varying concentrations of mucin and xanthan exhibited visual differences in color and opacity. Estimated T1 values were 2.23 ± 0.16, 2.37 ± 0.20, and 2.09 ± 0.18 s (mean ± standard deviation) for the three tubes with relative mucus concentrations of 1, 2.5, and 5, respectively. Corresponding T2 values were 806.6 ± 105.6, 687.4 ± 78.0, and 475.0 ± 41.3 ms, while diffusivity values were 2.36 ± 0.14, 2.31 ± 0.18, and 2.33 ± 0.18 µm^2^/ms for the three tubes. Only the T2 relaxation times showed a significant linear correlation with mucus concentration (*r* = -0.9998, *p* = 0.0112).

**Conclusion:**

UTE-DESS MRI could be feasible for characterizing airway mucus. T2 relaxation time showed a strong inverse correlation with increasing mucus concentration, indicating its sensitivity to mucus content.

**Key Points:**

***Question*** Can a novel quantitative ultrashort echo time MRI sequence noninvasively characterize airway mucus properties and overcome conventional lung imaging limitations in muco-obstructive diseases?

***Findings*** In this controlled *in vitro* model, UTE-DESS MRI mapping demonstrated that T2 relaxation times alone showed a strong inverse correlation with increasing mucus concentrations.

***Relevance statement*** This exploratory proof-of-concept establishes a technical foundation for radiation-free, noninvasive T2 mapping to monitor and phenotype chronic obstructive pulmonary disease and other mucoobstructive lung diseases in future in vivo studies.

**Graphical Abstract:**

**Figure:**
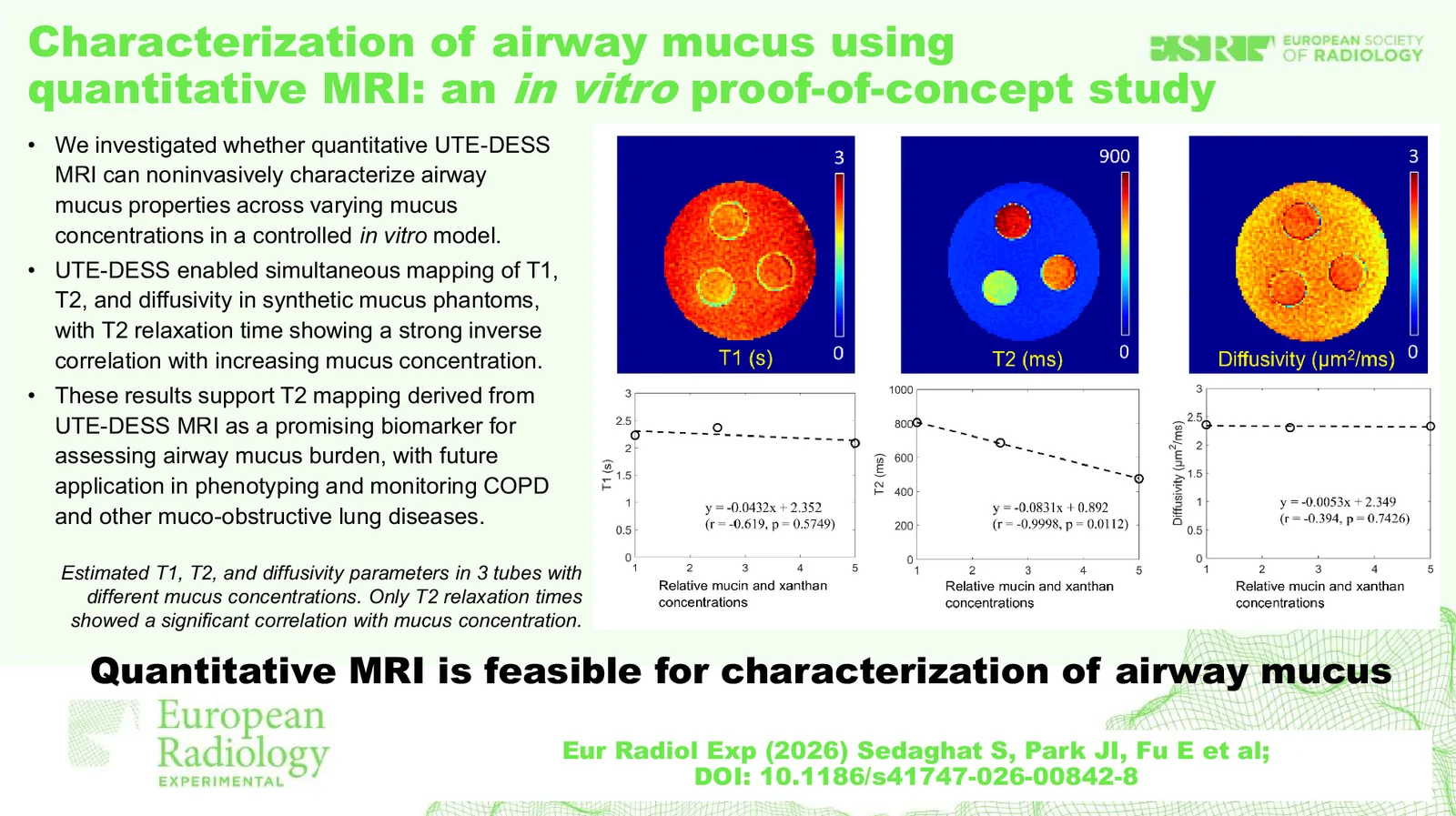

## Introduction

Chronic obstructive pulmonary disease (COPD) is a chronic airway disease characterized by persistent respiratory symptoms and progressive airflow limitation resulting from a combination of small airway remodeling, parenchymal destruction (emphysema), and loss of elastic recoil [1]. COPD is the fourth leading cause of death worldwide, responsible for approximately 3.5 million deaths in 2021 and accounting for 5% of all global deaths [2] and projected to reach 600 million cases by 2050 [3].

Oxidative stress and protease–antiprotease imbalance in COPD promote goblet cell hyperplasia and reduce ciliated cells, ultimately leading to both increased mucus production and impaired mucociliary clearance, creating a cycle of chronic inflammation and airway obstruction [4]. Airway mucus hypersecretion is a hallmark of COPD, contributing to mucus plug formation, which accelerates lung function decline and triggers acute exacerbations [5]. These changes impair the natural clearance mechanisms, reducing the airway’s defensive ability against pathogens, increasing the risk of airway damage and inflammation [4, 5].

Mucus plugs are emerging as potential markers for lung function impairment and exacerbation risk in COPD [6]. Previous studies have demonstrated a significant association between mucus plug score and reduced functional outcomes, including lower forced expiratory volume–FEV₁ and decreased peripheral oxygen saturation [6–10]. Mucus hypersecretion is considered a therapeutic target for chronic airway disease like COPD, presumably by reducing mucus plugging in airways [11].

Although bronchoscopy remains the gold standard for mucus visualization, it has several limitations, including its invasive nature, procedural risks, high cost, and limited ability to assess regions beyond the medium-sized bronchi [12]. Conventional magnetic resonance imaging (MRI), although well-established as the modality of choice for soft-tissue characterization, has inherent limitations in lung imaging due to the low proton density of pulmonary tissues and rapid signal decay caused by susceptibility effects at air–tissue interfaces. These factors result in a poor signal-to-noise ratio and limited image quality with conventional MRI sequences [13], restricting their utility for evaluating airway pathology and intraluminal contents such as mucus. Recent technical advancements have sought to overcome these challenges by enabling signal acquisition at extremely short echo times [14–16], thereby allowing visualization of tissues with no or low signal on conventional MRI.

Among these advances, ultrashort echo time (UTE) MRI has emerged as a promising approach for pulmonary MRI [16–18]. Building on this concept, we hypothesize that ultrashort echo time double-echo steady-state (UTE-DESS) [19–22], a further development of standard UTE, may provide detailed morphological information along with simultaneous quantitative mapping of relaxation and diffusion parameters in the lung. The sensitivity of UTE-DESS to relativity and water mobility suggests particular relevance for airway mucus characterization, as these properties are expected to be influenced by mucus concentration, hydration, and viscoelasticity.

In this proof-of-concept study, we aimed to characterize and quantify airway mucus by using quantitative UTE-DESS in a controlled *in vitro* synthetic mucus model [23]. By systematically evaluating UTE-DESS–derived quantitative parameters across varying mucus concentrations, this work seeks to establish a technical foundation for future *in vivo* studies and to support the development of noninvasive, radiation-free MRI biomarkers for assessing mucus burden in COPD and other muco-obstructive lung diseases.

## Materials and methods

### UTE-DESS

**Fig. 1:**
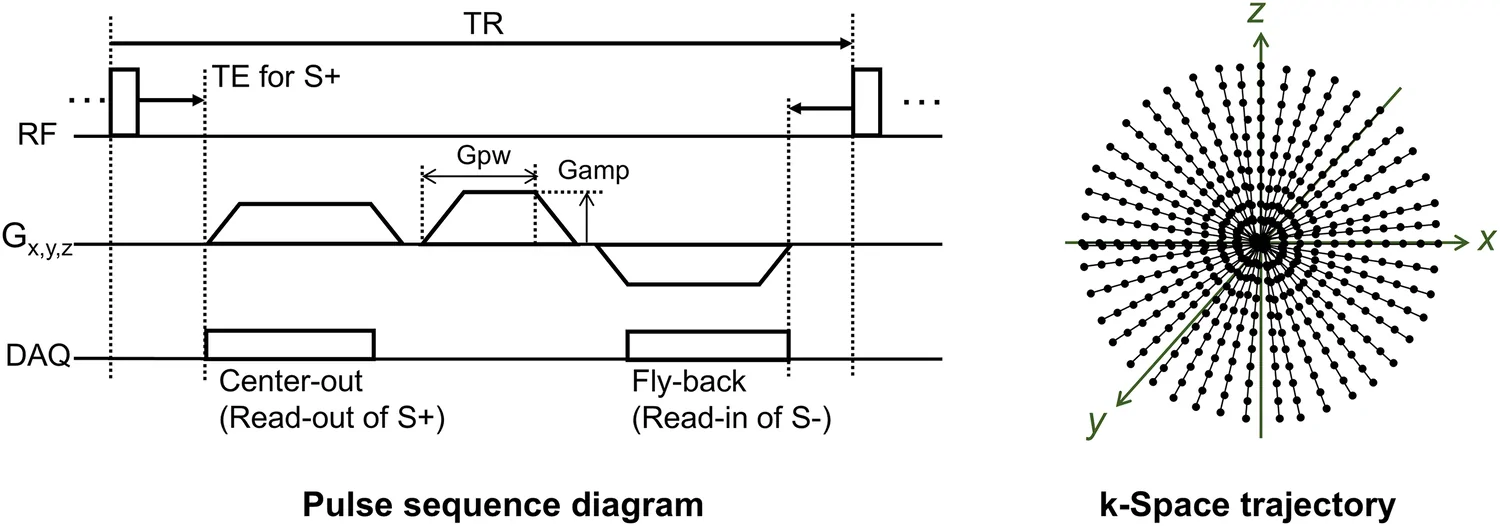
Pulse sequence diagram of UTE-DESS. S+ signal is mainly contributed by free induction decay, and S- signal is contributed by spin/stimulated echoes from previous RF excitations. A spoiler gradient is used to separate the signal into S+ and S-, as well as to add diffusion weighting. Higher gradient moment (GM, Gpw × Gamp) yields higher diffusion weighting. Gamp, Gradient amplitude; GM, Gradient moment; Gpw, Gradient pulse width; RF, Radiofrequency; S+, Free induction decay-like signal; S-, Spin/stimulated echo-like signal; UTE-DESS, Ultrashort echo time double-echo steady-state

UTE-DESS is based on steady-state free precession (SSFP) signal formation, which utilizes coherent signal buildup in various pathways through the steady state of transverse magnetization [19–22]. Figure 1 illustrates an MRI pulse sequence diagram of UTE-DESS. In this imaging technique, the repeated radiofrequency (RF) pulses with the coherent gradient (*i.e*., constant net gradient moment, GM, applied between RF pulses) establish steady state magnetization. Due to the non-zero GM, the SSFP signal is separated into two components, free induction decay-like S+ and spin/stimulated echo-like S-. S+ is observed immediately after the RF pulse, majorly contributed by UTE components, while S- is measured before the subsequent RF pulse, largely contributed by signal weighted by the combined spin-lattice relaxation time (T1), spin-spin relaxation time (T2), and diffusivity of water molecules.

The signal contrast of S+ and S- is influenced by the tissue’s MRI properties, such as proton density, T1, T2, and diffusivity, along with other imaging parameters. Based on this phenomenon, those quantities can be measured with repeated acquisition of UTE-DESS with changed scan parameters, achieving S+ and S- images with various signal weightings. For example, a variable flip angle (FA) scheme can be employed to achieve different T1 weightings. Different diffusion-weighting can be achieved by varying the GM, which is controlled by adjusting the gradient amplitude and/or gradient pulse width. T2 weighting can be achieved in S- echo. By fitting the acquired UTE-DESS S+ and S- signals (repeatedly measured with different FAs and GMs) to the SSFP signal model [24], we can simultaneously estimate quantitative MRI parameters, proton density, T1, T2, and diffusivity.

The quantitative estimation of T1, T2, and diffusivity *via* UTE-DESS relies on an established SSFP signal model. This specific framework and the associated pulse sequence parameters have been optimized through years of iterative development and prior measurement experience in short-T2 tissues.

### Mucus phantom

**Fig. 2:**
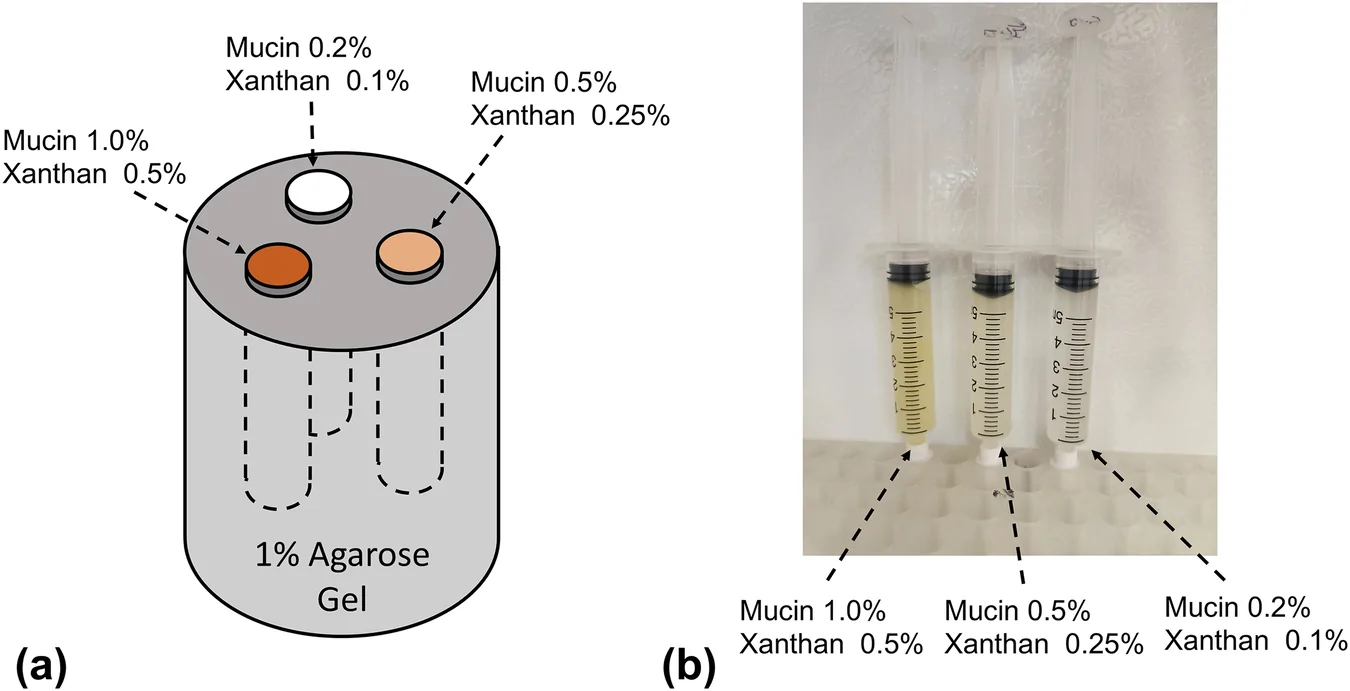
Synthetic mucus phantom. **a** Phantom design and (**b**) the manufactured phantoms. The synthetic mucus phantom was manufactured by mixing mucin and xanthan with varying concentrations

The synthetic mucin closely replicates the composition and viscoelastic properties of natural human laryngeal mucus and was formulated to fall within the physiological range reported in prior work [23]. Three synthetic mucus samples were prepared by mixing mucin (type III, bound sialic acid) at concentrations of 0.20%, 0.50%, and 1.0% with xanthan gum (XG) at concentrations of 0.10%, 0.25%, and 0.50%, following a recipe similar to that described by Peters et al [23] Each mucin solution was placed on an adjustable-speed rocker (Rocker II, Boekel Scientific, Feasterville, PA, USA) and mixed at 4 °C for 24 h to ensure complete dissolution and homogeneity. The corresponding concentrations of xanthan gum were then added, and the samples were mixed for an additional 24 h under the same refrigerated conditions to achieve stable viscoelastic properties. After thorough mixing, all samples were stored at 4 °C until imaging. Prior to MRI, the mucus solutions were transferred into syringes and embedded in 1% agar to provide mechanical support, prevent leakage, and ensure consistent positioning during scanning (Fig. 2a).

The concentrations (0.2–1.0% mucin and 0.1–0.5% XG) were increased in tandem to simulate the pathophysiological progression of mucus thickening in airway disease. This approach, informed by years of measurement experience with synthetic phantoms, models the increasing total polymer burden—the primary independent variable—to replicate the real-world macroscopic and viscoelastic changes observed in muco-obstructive conditions. Accordingly, we measured the “total relative mucus concentration” rather than the pure mucus concentration.

### Experimental setups

The mucus phantom was scanned using UTE-DESS MRI in a 3-T clinical scanner (MAGNETOM Prisma, Siemens Healthineers, Erlangen, Germany). A receive-only 32-channel head-coil was used. The MRI parameters for UTE-DESS were as follows: FA = 10° or 30°; repetition time = 15 ms; echo time of S+ = 0.03 ms; gradient amplitude = 6 or 24 mT/m; gradient pulse width = 5 ms; field-of-view = 200 × 200 × 192 mm^3^; matrix size = 400 × 400 × 400, readout bandwidth = 113.6 kHz; and scan time = 225 s/scan (four scans with the two FAs and two gradient amplitudes). A vendor-provided B_1_ mapping sequence was additionally applied to estimate the actual flip angle, with an approximate scan time of 20 s.

The phantom imaging was performed at an ambient room temperature of approximately 20 °C. To ensure thermal stability prior to acquisition, the phantom was allowed to equilibrate at room temperature for 2 h.

### Statistical analysis

Quantitative MRI parameters (T1, T2, and diffusivity) were reported as mean ± standard deviation for each synthetic mucus sample. Linear regression and Pearson correlation coefficients (*r*) were calculated to assess the relationship between quantitative MRI parameters and the relative mucus concentrations. The three concentration levels (ratio 1:2.5:5) were selected based on established expertise to represent the spectrum of mucus thickening in airway disease. Corresponding *p*-values were calculated to assess the strength and statistical significance of these associations. A *p*-value < 0.05 was considered statistically significant. All statistical analyses were performed using SPSS Statistics v28.0 (IBM, Armonk, NY, USA) and Python v3.12.

## Results

### Visual examination

The three tubes exhibited visual differences in color and opacity, with increasing mucus concentration resulting in a progressively darker appearance and reduced transparency. Figure 2b shows the manufactured mucus phantoms with varying concentrations of mucin and xanthan (ratio = 1:2.5:5), reflecting the expected macroscopic changes associated with higher polymer content and increased solution density. These visual differences qualitatively confirm the successful preparation of phantoms with distinct mucus concentrations prior to MRI experiments.

### Technical results

**Fig. 3:**
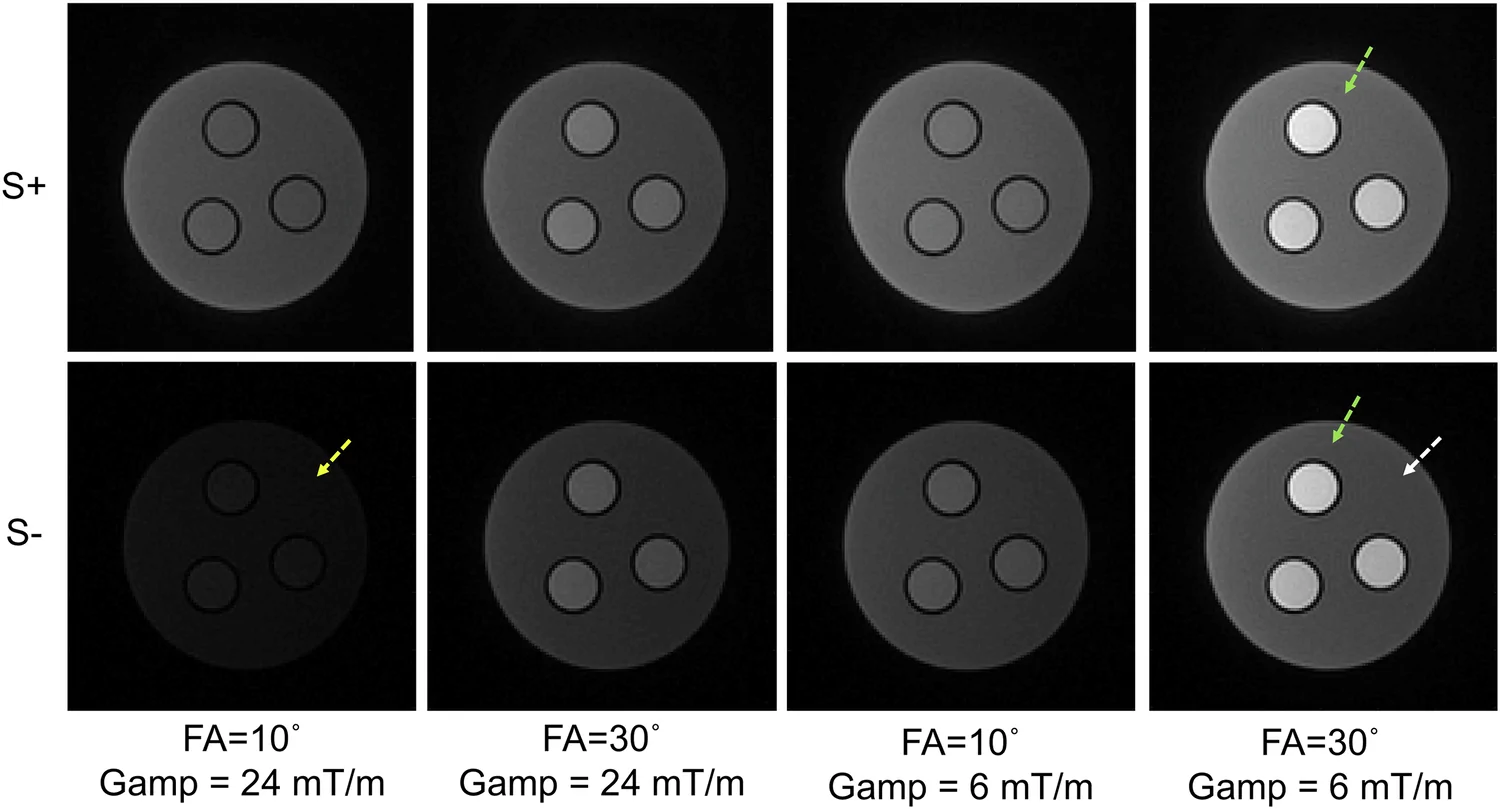
UTE-DESS images with different flip angles and gradient amplitude. The total 8 images from 4 scans, with different T1 (highlighted by the green arrows), T2 (highlighted by the white arrow), and diffusion weighting (highlighted by the yellow arrow), are used to simultaneously fit the T1, T2, and diffusivity parameters based on the SSFP signal model. SSFP, Steady-state free precession; UTE-DESS, Ultrashort echo time double-echo steady-state

Figure 3 shows the acquired S+ and S- images from UTE-DESS acquisitions with two different FAs (10° and 30°) and two different GMs (30 and 120 mT/m·ms). Overall, differences in T2 weighting are observed between the S+ and S- echoes. With lower GM and higher FA, the contrast between the S+ and S- signals is largely dominated by T2 relaxation, as indicated by the white arrow. Acquisition with higher FA (30°) results in stronger T1 weighting in both S+ and S- signals, as indicated by the green arrows. The S+ signal is minimally affected by diffusion weighting, whereas the S- signal exhibits varying diffusion weighting depending on GM, with higher GM (120 mT/m·ms) producing stronger diffusion weighting. Notably, the combination of lower FA (10°) and higher GM with a gradient amplitude of 24 mT/m resulted in the strongest diffusion weighting, as indicated by the yellow arrow, presumably due to longer signal pathways required to form the echo, effectively mimicking a long mixing time in conventional diffusion-weighted imaging.

### Measured parameters

**Fig. 4:**
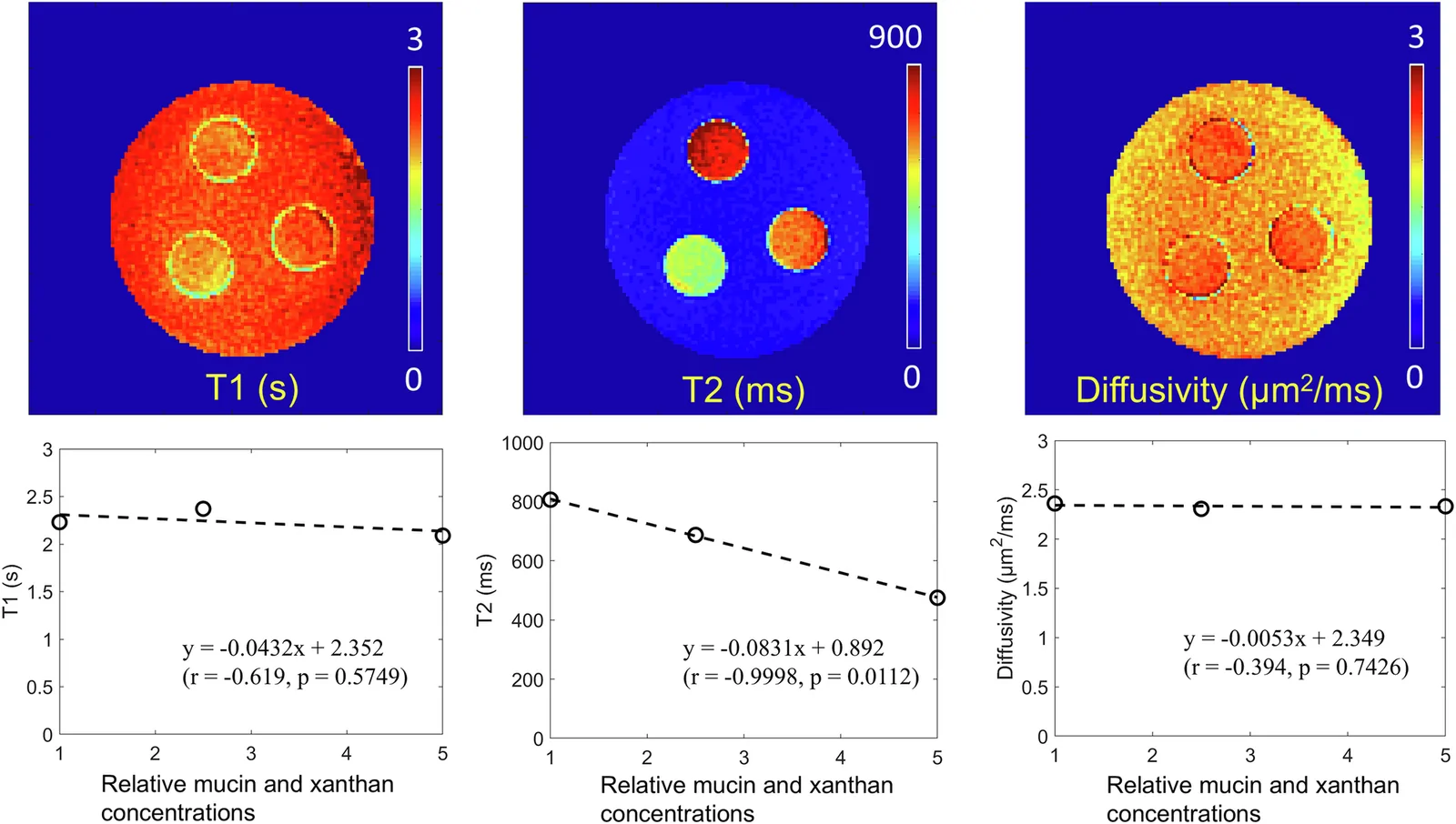
Estimated T1, T2, and diffusivity parameters in three tubes with different mucus concentrations. Only T2 relaxation times showed a significant correlation with the synthetic mucus concentration

Figure 4 demonstrates the fitted parameters using UTE-DESS. The estimated T1 values were 2.23 ± 0.16, 2.37 ± 0.20, and 2.09 ± 0.18 s for the three tubes with relative mucus concentrations of 1, 2.5, and 5, respectively. The estimated T2 values were 806.6 ± 105.6, 687.4 ± 78.0, and 475.0 ± 41.3 ms for the three tubes with relative mucus concentrations of 1, 2.5, and 5, respectively. The estimated diffusivity values were 2.36 ± 0.14, 2.31 ± 0.18, and 2.33 ± 0.18 µm^2^/ms for the three tubes with relative mucus concentrations of 1, 2.5, and 5, respectively. Only the T2 relaxation times showed a significant linear correlation with the total relative mucus concentration (*r* = -0.9998, *p* = 0.0112).

## Discussion

In this study, we evaluated the feasibility of the UTE-DESS MRI sequence for quantitative characterization of airway mucus using a controlled *in vitro* phantom model.

Our results demonstrate that UTE-DESS could enable simultaneous mapping of multiple quantitative MRI parameters, with T2 relaxation time showing a strong and significant correlation with increasing mucus concentration. These findings suggest that UTE-DESS–derived T2 measurements are particularly sensitive to variations in mucus content and may serve as a potential quantitative imaging biomarker for mucus burden. While these exploratory findings are highly encouraging, given the limited number of concentration levels evaluated, this work should be considered a proof-of-concept study rather than a fully validated clinical imaging methodology.

This study highlights the potential of UTE-DESS MRI as a noninvasive, radiation-free tool for assessing airway mucus burden. Most previous imaging studies of airway mucus in COPD have focused on chest computed tomography (CT) rather than MRI. CT offers high spatial resolution and wide availability, allowing direct visualization and quantification of mucus plugs, airway wall thickness, and emphysema, particularly in medium-sized airways. In contrast, MRI has historically been less suitable for assessing airway mucus because of technical limitations and lower sensitivity to airway structural abnormalities. As a result, CT has become the primary imaging modality for studying mucus plugging in COPD [25–27]. However, quantitative evaluation of mucus is clinically relevant in COPD, as mucus plugging is increasingly recognized as a critical and independent pathological feature associated with adverse patient outcomes. Mucus plugs, often detected in medium-to-large-sized airways, function as a crucial imaging biomarker with profound prognostic implications [25, 28].

Occlusion of airways by mucus plugging may increase susceptibility to infection by impairing ventilation, limiting oxygen diffusion, and creating regions of local hypoxia that favor microbial growth. When mucus plugs involve multiple bronchi, they can substantially disrupt airway clearance, thereby increasing the risk of pneumonia and triggering COPD exacerbations. In addition, mucus plugging contributes to ventilation–perfusion mismatch by obstructing airflow to otherwise perfused lung regions, thereby impairing gas exchange. Over time, this physiological derangement may progress to hypoxemia and respiratory failure, which remains a leading cause of morbidity and mortality in patients with COPD [11, 29–31].

MRI is rarely used in routine structural or functional lung evaluation, but UTE pulse sequences have recently emerged as promising approaches for direct and efficient lung visualization [17, 18, 32]. These sequences allow imaging of tissues with very short T2 relaxation times, which are not adequately visualized with conventional MRI [33]. UTE-MRI with respiratory-gating has demonstrated better contrast for mucus plugs due to their inherent sensitivity to fluid [34]. Although mucus is not a short T2 substance, UTE-MRI can be beneficial due to its strong susceptibility in the airway environment. Recent studies have demonstrated that UTE-MRI provides diagnostic performance comparable to high-resolution CT, while avoiding ionizing radiation, making it a superior imaging modality [35–37]. The further development of conventional UTE-MRI and UTE-DESS MRI may offer a valuable, radiation-sparing method to precisely phenotype COPD patients and visualize a key structural abnormality that drives patient morbidity and mortality. By prospectively applying the sequence to COPD patients, we expect that the S+ of UTE-DESS will provide morphological information about lung tissues, while the S- (T2-weighted) and fitted T2 parameters will provide information about mucus. By enabling the direct visualization of mucus and other lung tissues, UTE-MRI may provide a more comprehensive understanding of mucus dynamics, facilitating better assessment of disease severity, monitoring of therapeutic responses, and potentially predicting exacerbations in COPD patients. While mucus plugging is a major contributor to ventilation–perfusion mismatch and acute exacerbations, it exists within a complex pathophysiological landscape that includes airway wall thickening and emphysematous tissue loss. However, our study focused on this important aspect of ventilation–perfusion mismatch, as the direct visualization and quantification of mucus on MRI are rarely described in the literature.

The scientific literature describes a variety of formulations for producing synthetic mucus, which offers a less invasive, more reproducible, and cost-effective alternative to using native mucin [38]. Among the available materials, XG, an exopolysaccharide widely used in the food and pharmaceutical industries, is particularly attractive because of its well-characterized rheological properties, including high viscosity, shear-thinning (pseudoplastic) behavior, and stability in aqueous solutions [39, 40]. These properties allow XG-based gels to closely mimic the viscoelastic behavior of airway mucus while enabling precise control over composition and concentration. In this study, we use XG as the primary component to generate three synthetic mucin samples with distinct rheological characteristics, which serve as controlled phantoms for validating and characterizing the performance of the UTE-DESS imaging method.

In this model, mucin and xanthan gum concentrations were varied simultaneously to maintain stable viscoelastic properties representative of human mucus. Consequently, it is not possible to distinguish the individual effects of mucin *versus* xanthan gum on T2 relaxation. However, from a clinical perspective, the total solid-to-liquid ratio is the primary driver of mucus plugging and disease severity. Therefore, the sensitivity of T2 to the total polymer burden remains a highly relevant finding for monitoring muco-obstructive diseases.

Although this study was performed *in vitro*, the demonstrated sensitivity of UTE-DESS to mucus properties establishes an important foundation for future *in vivo* validation and clinical translation in patients with COPD and other muco-obstructive lung diseases. Future work will focus on advancing the clinical application of the UTE-DESS sequence. First, technical optimization will address current limitations related to acquisition time and motion artifacts by incorporating accelerated imaging approaches, such as compressed sensing and parallel imaging, along with motion-compensation strategies including respiratory gating, to enable rapid and robust data acquisition in human subjects. Second, following technical validation, the sequence will be applied in cohorts of patients with COPD and healthy controls to evaluate its utility as a noninvasive imaging biomarker for disease staging and for monitoring therapeutic response. Here, both *in vivo* examination and an *ex vivo* study with human sputum will take place. Finally, the scope will be expanded to investigate the applicability of UTE-DESS across a broader range of pulmonary pathologies, including cystic fibrosis and other bronchiectatic diseases, with the goal of providing new imaging insights into the spectrum of chronic lung diseases.

Finally, we did not incorporate any reference standard analysis with a direct comparison to other sequences. Conventional MRI sequences are fundamentally limited in lung imaging due to the high density of air–tissue interfaces. These interfaces create massive magnetic susceptibility gradients that cause rapid signal decay, rendering the lung parenchyma largely invisible. Consequently, quantitative lung MRI using conventional sequences is not well-established, resulting in a lack of validated, imaging-based reference standards for comparison. The primary objective of this study was to establish a foundation for mucus-specific characterization rather than absolute physical calibration. Future studies could involve comparative analyses against other state-of-the-art MRI sequences.

This study has several limitations. First, the phantom model does not fully replicate the biochemical and biophysical properties of native airway mucus, such as variations in viscosity, hydration state, and chemical composition [41]. This may limit the direct translation of results to *in vivo* settings. Second, the absence of respiratory motion in the phantom reduces applicability to clinical lung imaging, where breathing and cardiac motion can cause blurring and artifacts [42]. Motion-compensated techniques (*e.g*., respiratory gating or navigator echoes) may be more useful to translate these results into real-world settings [43, 44]. Additionally, the linear correlation analysis was based on only three concentration levels. Consequently, this statistical interpretation remains highly exploratory, despite the encouraging near-perfect linearity observed. Future studies utilizing a much larger array of sample concentrations, varied biochemical compositions, and blind testing are strictly required to transition this framework from a proof-of-concept into a validated methodology. Furthermore, we acknowledge the lack of intra- and inter-session repeatability data as a limitation of the current study design. As this initial investigation focused on proof-of-concept and baseline parameter estimation, test-retest acquisitions were not performed. Without this assessment, the ultimate robustness of the estimated parameters remains to be fully established. Future studies will need to incorporate dedicated repeatability protocols to comprehensively validate the quantitative reliability of this technique. Finally, the relatively long UTE-DESS acquisition time compared to CT may restrict clinical feasibility, particularly in patients with severe COPD [19]. Technical improvements, such as parallel imaging, compressed sensing, and advanced reconstruction algorithms, could help shorten acquisition time while maintaining diagnostic image quality.

## Conclusion

In conclusion, this proof-of-concept *in vitro* study demonstrates that UTE-DESS MRI could be feasible for quantitative characterization of airway mucus. Among the measured parameters, T2 relaxation time showed a strong inverse correlation with increasing mucus concentration, indicating its potential sensitivity to mucus content. By providing both morphological and quantitative information without ionizing radiation, UTE-DESS MRI could be a promising noninvasive tool for assessing airway mucus burden. These findings establish a foundation for future *in vivo* studies.

## Data Availability

The data and materials that support the findings of this study are available from the corresponding author upon reasonable request.

## Abbreviations

COPD: Chronic obstructive pulmonary disease
CT: Computed tomography
FA: Flip angle
GM: Gradient moment
MRI: Magnetic resonance imaging
RF: Radiofrequency
S-: Spin/stimulated echo-like signal
S+: Free induction decay-like signal
SSFP: Steady-state free precession
UTE: Ultrashort echo time
UTE-DESS: Ultrashort echo time double-echo steady-state
XG: Xanthan gum
